# Function and Application of the CRISPR-Cas System in the Plant Pathogen *Erwinia amylovora*

**DOI:** 10.1128/aem.02513-21

**Published:** 2022-03-14

**Authors:** Michael Parcey, Steven Gayder, Alan J. Castle, Antonet M. Svircev

**Affiliations:** a Centre for Biotechnology, Brock University, St. Catharines, Ontario, Canada; b Agriculture and Agri-Food Canada, Vineland Station, Ontario, Canada; c Institute of Food and Beverage Innovation, Zurich University of Applied Sciences, Wädenswil, Switzerland; d Department of Biological Sciences, Brock University, St. Catharines, Ontario, Canada; University of Tennessee at Knoxville

**Keywords:** CRISPR-Cas, phage biocontrol, plant pathogens, phage resistance, bacteriophage therapy, antibiotic resistance

## Abstract

Phage-based biocontrol is an emerging method for managing the plant pathogen Erwinia amylovora. Control of E. amylovora in North America is achieved chiefly through the application of streptomycin and has led to the development of streptomycin resistance. Resistant E. amylovora can be tracked through the analysis of CRISPR spacer sequences. An alternative to antibiotics are bacterial viruses, known as phages, which lyse their hosts during replication to control the bacterial population. Endogenous CRISPR-Cas systems act as phage resistance mechanisms however, preliminary genomic analysis suggests this activity is limited in E. amylovora. This leaves the functionality of the CRISPR-Cas system, any clade-based differences, and the impact which this system may have on phage-based biocontrol in question. In this study, the CRISPR arrays from 127 newly available genomic sequences of E. amylovora were analyzed through a novel bioinformatic pipeline. Through this, the Eastern and Western North American clades were shown to be incompatible with the current PCR-based approaches for tracking E. amylovora given the size and composition of their CRISPR arrays. Two artificial CRISPR arrays were designed to investigate the functionality of the CRISPR-Cas system in E. amylovora. This system was capable of curing a targeted plasmid and providing phage resistance but was not the source of phage resistance observed within the controls. This suggests that while the CRISPR-Cas system is an important defense mechanism for invasive plasmids, an as yet unidentified mechanism is the primary source of phage resistance in E. amylovora.

**IMPORTANCE** Erwinia amylovora is an economically significant agricultural pathogen found throughout the world. In North America, E. amylovora has developed streptomycin resistance and therefore alternative treatments using phages have received increased attention. In this study, we analyzed recently published genomes to determine that two significant groups of E. amylovora are poorly identified using the current, CRISPR-based tracking methods. We also showed that the CRISPR-Cas system and an unidentified mechanism work together to provide a significant degree of resistance against one of the phages proposed for phage-based biocontrol.

## INTRODUCTION

CRISPR-Cas is one of the most important systems in the modern study of Erwinia amylovora. It has become a popular tool for tracking the dissemination of the pathogen, monitoring the prevalence of antibiotic resistance, and can significantly impact the development of phage-mediated biological control (phage biocontrol) (1–5). Despite this, little is known about the activity of the CRISPR-Cas system in E. amylovora or how this activity may impact these applications.

E. amylovora, the causal agent of fire blight disease, is an destructive agricultural plant pathogen that infects plants such as apples and pears belonging to the *Amygdaloideae* subfamily (6). There are three major *Amygdaloideae*-infecting clades of E. amylovora, all of which originate from North America. The Widely Prevalent (WP) clade, the most commonly isolated group of E. amylovora, was disseminated globally through the movement of infected material in the early to mid 1900s (3, 6). The Eastern North American (ENA) and Western North American (WNA) clades are found strictly in the continental USA and Canada in the eastern and western pome fruit growing regions, respectively (7, 8). In the USA and Canada, control of E. amylovora infection is achieved through the application of the antibiotic streptomycin in spring (9). This high antibiotic pressure has resulted in the development of streptomycin resistance (SmR) in the E. amylovora population, particularly prevalent in the WNA clade (2, 7). Subsequently, research into alternatives to antibiotics, such as phage biocontrol, has increased (10).

Monitoring the spread of E. amylovora utilizes the spacer acquisition function of the CRISPR-Cas system. Through spacer acquisition, the bacterium gains DNA sequences from invasive mobile genetic elements (iMGEs), such as phages and plasmids, which have a negative impact on survival. These sequences, termed spacers, are inserted sequentially between **c**lustered, **r**egularly **i**nterspaced, **s**hort **p**alindromic **r**epeats (CRISPR) to form CRISPR arrays (11). E. amylovora has two intact CRISPR arrays, CRR1 and CRR2 (also known as CR1 and CR2), which flank the Type I-E CRISPR-associated (Cas) gene cluster. In addition, E. amylovora has a third CRISPR array, CRR4 (synonym CR3), which is a remnant of the Type I-F CRISPR-Cas system found in other members of the genus such as *Erwinia pyrofoliae* and Erwinia tasmaniensis (1, 12). The presence and order of CRISPR spacers creates a heritable record of significant iMGE pressures within the bacterium’s environment and creates a pattern by which phylogeny can be elucidated (1, 2). This technique has been used extensively to describe the WP clade across Eurasia, Portugal, and the northeastern USA (3–5, 13). PCR-based techniques can effectively describe the WP clade and can be used to create the observed CRISPR profiles because of the small size and high level of conservation found within those CRISPR arrays. However, these profiles are poorly defined within newly described ENA and WNA clades.

Target interference is the function of the CRISPR-Cas system which provides phage resistance and has been exploited to generate novel gene-editing technologies (14, 15). CRISPR spacers, promoted by an upstream leader sequence, are transcribed and processed into CRISPR RNA (crRNA). In the Type I-E system found in E. amylovora, this is completed by Cas6e. The Cas6e-bound crRNA and the interference-associated Cas proteins assemble the **C**RISPR-**as**sociated **c**omplex for **a**ntiviral **de**fense (Cascade) (16). When Cascade encounters a DNA sequence complementary to the bound crRNA (protospacer), with an upstream PAM element (protospacer adjacent motif), Cascade will recruit Cas3 to cleave and degrade the dsDNA of the iMGE (17, 18). In the context of phage biocontrol, this provides a mechanism by which E. amylovora may develop or have inherent phage resistance. Previous studies on the CRISPR-Cas system of E. amylovora have shown isolates which harbored the plasmid pEU30 can also have CRISPR spacers to pEU30 (1, 12). This contradiction led Rezzonico et al. (2011) to postulate that some of the CRISPR-Cas systems of E. amylovora were nonfunctional or at least incapable of interference.

In order for the CRISPR-Cas system to act as a monitoring tool, a relationship between the major *Amygdaloideae*-infecting clades and CRISPR arrays needs to be established (1–5). The level of CRISPR-Cas activity within E. amylovora also needs to be evaluated to determine the risk of resistance or immunity to phage biocontrol. In this study, the CRISPR arrays from 127 newly available genomic sequences of E. amylovora were analyzed through bioinformatics to produce a phylogeny based on presence and arrangement of the CRR1, CRR2, and CRR4 CRISPR arrays. The unique CRISPR spacers were compared to known iMGEs to identify the genetic origin. As the activity of the CRISPR-Cas system has never been experimentally demonstrated in E. amylovora, two synthetic CRISPR arrays to *Erwinia* phage ΦEa21-4 were developed. These artificial CRISPR arrays were transformed into isolates representing the major *Amygdaloideae*-infecting clades. The resistance to transformation with an engineered plasmid was established as a control. Subsequently, infection by phage ΦEa21-4 was therefore tested in the presence of an active CRISPR-Cas background.

## RESULTS AND DISCUSSION

### The CRISPR groups of *Amygdaloideae-*infecting E. amylovora.

**FIG 1:**
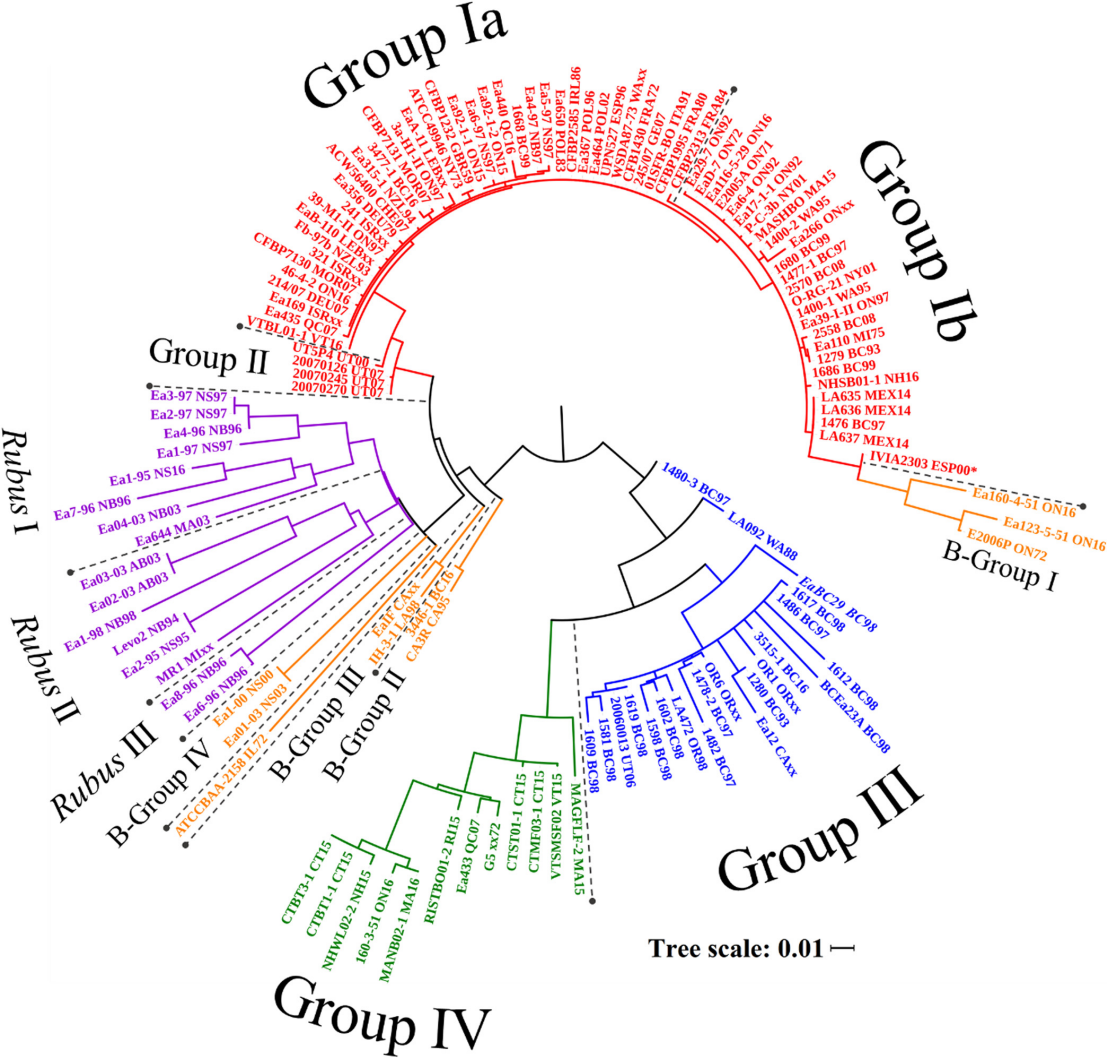
Phylogenetic analysis of E. amylovora based on the presence and order of CRR1, CRR2, and CRR4 spacers. The phylogeny has a bootstrap value of 1000 and was visualized in iTOL with a cutoff of 70%. colors indicate the phylogenetic clade, as previously determined through chromosomal analysis (7), associated with each isolate and the labels indicating the associated CRISPR group: red, widely prevalent; blue, western North American; green, eastern North American; orange, B-Group; purple, *Rubus*-infecting. Isolate IVIA2303(*) had a truncated CRR2 array attributed to poor sequence sequencing.

CRISPR spacers arrangements were among the first methodologies used for genotyping of E. amylovora (1, 12, 19). In this study, a novel bioinformatic pipeline was developed in Biopython to identify E. amylovora CRISPR spacers and to form a species-wide consensus pattern for CRR1, CRR2, and CRR4. A total of 10,829 spacers were identified using this pipeline with 1,000 unique spacer sequences. From the presence or absence of the CRISPR spacers relative to this consensus, a phylogeny was produced (Fig. 1). This phylogeny is in strong agreement with the clade development resolved using whole chromosomal or plasmid pEA29 sequences (7, 8). The major *Amygdaloideae-*infecting (AI) clades are composed of CRISPR groups Ia, Ib, II, III, and IV, with the Widely Prevalent clade comprising the Ia, Ib, and II as previously described (Fig. 1, red) (12).

Since the late 18th century, the WP clade has spread from New York State to nearly all apple producing countries and currently accounts for all sequenced E. amylovora isolates found outside North America (3, 7). These non-North American isolates are found exclusively within group Ia. Group Ia, with the exception of Ea440 and CFBP2313, has a larger CRR2 array which contains 31 to 35 spacers whereas group Ib has 21 to 26 (Table 1). Both group Ia and group Ib have 27 to 35 spacers in the CRR1 array. Group II is composed of 4 isolates from UT, USA and is distinct from group Ia and group Ib via the CRR1 array which contains only 12 to 19 spacers. Only 5 isolates were found to have nonsynonymous SNPs within the Cas genes of group I and group II. Therefore, despite the differences in the CRISPR arrays, the activity of the CRISPR-Cas system is predicted to be the same across this clade.

**TABLE 1:** Size of CRISPR arrays found in E. amylovora

CRISPR group	CRR1 spacers			CRR2 spacers			CRR4 spacers		
	Unique	Min	Max	Unique	Min	Max	Unique	Min	Max
*Amygdaloideae*-infecting	152	12	110	67	21	58	5	5	5
Group Ia	36	28	36	35	31^*a*^	35	5	5	5
Group Ib	36	27	36	26	21	26	5	5	5
Group II	19	12	19	35	27	35	5	5	5
Group I & II	40	12	36	35	21	35	5	5	5
Group III	119	44	110	52	25	49	5	5	5
Group IV	96	53	95	59	25	58	5	5	5
B-Group	166	18	53	178	22	49	5	5	5
B-Group I	39	29	31	45	22	23	5	5	5
B-Group II	22	18	22	52	49	49	5	5	5
B-Group III	29	18	23	41	36	39	5	5	5
B-Group IV	42	42	42	32	32	32	5	5	5
ATCC-BAA2158	53	53	53	38	38	38	5	5	5
*Rubus*-infecting	347	20	55	178	16	40	6	3	6
Rubus I	147	20	41	70	16	38	6	3	6
Rubus II	172	48	55	83	31	40	6	5	6
Rubus III	52	51	51	30	28	30	6	4	6
E. amylovora	624	12	110	370	16	58	6	3	6

a Excluding Ea440 (27 Spacers) and CFBP2313 (29 Spacers)

WNA and ENA are two AI clades unique to North America and form the distinct CRISPR group III (Fig. 1, blue) and group IV (Fig. 1, green), respectively. These two CRISPR groups have the largest arrays, with the maximum size of 110 spacers and 58 spacer for the CRR1 and CRR2 arrays, respectively. Despite the size of these CRISPR arrays, group III and group IV share many spacers absent in the WP clade which draws new connections between the WNA and ENA clades. It was previously observed that the WP, WNA, and ENA clades were of equal genetic variance relative to one another when observed through whole-genome sequence alignment (7, 8). However, phylogenies produced using plasmid pEA29 and the CRISPR arrays suggest the WNA clade is ancestral to the ENA clade (7). This is supported by further analysis of the genetic region located between the CRR1 array and the Cas genes (1): this 2361 bp sequence is composed of three putative genes present in WP isolates but is entirely absent in all ENA and WNA isolates.

Another similarity between the ENA and WNA clades is the shared 63 bp CRR1 spacer first identified in WNA isolates (12). The average size of the CRISPR spacers found in this and previous studies is 32 bp (1, 12). The elongated spacer found in the WNA clade is thought to have formed from the partial deletion of the subsequent repeat/spacer and bears a G18A transition in ENA isolates. An unfortunate reality of draft genome analysis from short-read NGS is an increased number of sequence assembly errors within repetitive genomic regions such as CRISPR arrays (20). As such, the other 33 potential larger spacers were not included within the phylogenetic analysis as they need to be confirmed through further sequencing. It should be noted however that significantly more of these elongated CRISPR spacers may exist within the E. amylovora population.

Recently, CRISPR spacer analysis was used to track the dissemination of E. amylovora across Eurasia, Portugal, and New York State. PCRs were designed to amplify either the entire CRISPR array (4, 5) or a spacer, designated 1029, which had duplicated within the CRR1 array (3). Both of these methodologies are capable of a high level of differentiation within the WP clade. Unfortunately, PCR amplification of the entire CRR1 and CRR2 arrays for the ENA and WNA clades is not a viable strategy as they can contain 3-fold more spacers and a resulting amplicon of over 3000 bp. Spacer 1029 was also absent from both the ENA and WNA. In fact, spacer 1029 and its duplicate were noticeably absent from the majority (42 of 68) WP genomes in this study. This method has previously been used to differentiate most of the tested Eurasian isolates however, only 68.1% and 22.7% of the WP genomes from Eurasia and North America, respectively, found in this study contained spacer 1029. While this may be due to the shortcomings of NGS as previously noted, the presence of spacer 1029 did not correlate with group Ia and Ib development. This would suggest that while spacer 1029 can be used to track group Ia across Europe, it may not be representative of the major difference within the WP clade, vis-à-vis the CRR2 array.

### CRISPR groups of the *Rubus*-infecting and B-Group superclades of E. amylovora.

The B-Group superclade is composed of several, highly distinct strains of E. amylovora which infect pome fruit but are genetically dissimilar to the WP, ENA, and WNA clades (7). The CRISPR arrays of these isolates form four unique CRISPR groups designated B-group I to IV (Fig. 1, orange) and are composed almost exclusively of unique spacers (Table 1). Isolate ATCC BAA-2158 for example, shares only two CRISPR spacers with any other group. The exception to this is B-group I which shares many of its CRR1 spacers with group I. This is an interesting contrast to the whole-genome or pEA29 comparisons which showed little relation between the WP isolates and B-Group I. It may therefore represent a unique divergent event of B-group I from the WP clade (7).

Certain strains of E. amylovora are also capable of infecting *Rubus* plants (eg. raspberry) during sporadic outbreaks however, these isolates lack the sorbitol dehydrogenase operon required to infect *Amygdaloideae* plants (21, 22). The highest level of phylogenetic differentiation through the CRISPR arrays occurs in the *Rubus*-infecting (RI) isolates of E. amylovora. These isolates were broken into three groups, *Rubus* I to III, for characterization (Fig. 1, purple). The CRR1 array of *Rubus* I is the smallest with 20 to 41 spacers while *Rubus* II and *Rubus* III have 48 to 55 (Table 1). All three groups have CRR2 arrays ranging from 16 to 40 spacers. Despite the similarity in size, the *Rubus* CRISPR groups were distinct from one another, sharing only 29 of the 554 unique CRR1 and CRR2 spacers.

The CRR4 array is associated with the Type1-F Cas system present in *E. pyrifoliae* and E. tasmaniensis but absent in E. amylovora (1, 12). Therefore, while the CRR1 and CRR2 arrays can be differentiated by both the acquisition and loss of CRISPR spacers, CRR4 would be differentiated exclusively by loss. The CRR4 array of both the AI strains and the B-Group is five spacers in length. One distinct feature of the *Rubus* CRR4 arrays is the presence of a 6th unique CRR4 spacer (Table 1). Like the others, B-Group I, B-group IV and ATCC BAA-2158 have five spacers; however, these isolates lost the last CRR4 spacer of the AI isolates and maintained the CRR4 spacer unique to RI isolates. There is also 3 SNPs which distinguish the AI isolates and the B-group from the *Rubus* superclade, two located in the third to last and one in the last CRR4 spacer (3′ to 5′). These SNP differences were observed across all sequences in this study, suggesting the loss of CRR4 spacers in the AI and B-Group relative to the *Rubus*-infecting groups occurred independently.

### The genetic sources of E. amylovora CRISPR spacers.

While the presence of CRISPR spacers is used to determine the lineage of E. amylovora and track its dissemination globally, CRISPR spacers also give insight into predaceous environmental pressures (17). Each spacer found within the CRISPR array is identical to another sequence, known as a protospacer, found within antagonistic iMGEs (11). The majority of spacers within the CRISPR system of E. amylovora, which have a sequenced genetic source, originate from plasmid pEU30 and were acquired by CRISPR groups III and IV (Table 2), concurring with previous observations (12). Indeed, this is expected as pEU30 is most commonly found in WNA isolates and therefore resistance is most likely to develop within that clade (7). Plasmids pEU30 and pEA72 share an ∼3000 bp region which encompasses nearly half of all CRISPR spacers capable of targeting pEA72. Surprisingly, group IV had very few spacers specific to pEA72 despite the ENA isolates being the primary source of this plasmid within available genomes (7). CRISPR groups I and II have 75 unique spacers found in the CRR1 and CRR2 arrays however only 16 match protospacers in published genetic sequences: half of which are to plasmids from other genera such as Escherichia and Klebsiella.

**TABLE 2:** Protospacers targeted by the CRR1 and CRR2 CRISPR arrays of E. amylovora

CRISPR group	CRISPR spacers (CRR1 : CRR2)							
	Plasmid origin				Phage origin			
	pEU30	*Erwinia* spp.	Other	Total	ΦEt88	*Erwinia* spp.	Other	Total
*Amygdaloideae*-infecting	40 : 16	4 : 3	11 : 3	55 : 22	3 : 0	0 : 0	2 : 0	5 : 0
Group I & II	2 : 0	1 : 3	5 : 3	8 : 6	2 : 0	0 : 0	0 : 0	2 : 0
Group III	31 : 6	3 : 2	9 : 3	43 : 11	3 : 0	0 : 0	2 : 0	5 : 0
Group IV	32 : 15	2 : 2	7 : 3	41 : 20	1 : 0	0 : 0	2 : 0	3 : 0
B-Group	3 : 11	8 : 12	14 : 9	25 : 32	3 : 3	1 : 1	3 : 0	7 : 4
*Rubus*-infecting	3 : 1	14 : 10	4 : 9	21 : 20	19 : 9	1 : 1	5 : 7	25 : 17
*Rubus* I	1 : 1	3 : 2	4 : 2	8 : 5	9 : 3	0 : 1	6 : 5	15 : 8
*Rubus* II	2 : 0	10 : 7	1 : 3	13 : 10	10 : 5	1 : 0	6 : 1	17 : 6
*Rubus* III	3 : 1	14 : 10	4 : 9	21 : 20	19 : 9	1 : 1	5 : 7	25 : 17
*E. amylovora*	43 : 23	24 : 14	31 : 21	98 : 68	27 : 16	4 : 3	24 : 9	55 : 28

One of the most striking differences between the AI and RI superclades is the difference in protospacer targets. The AI superclade has 77 CRISPR spacers to plasmids and only five spacers to known phages. In contrast, the RI superclade has 41 spacers to plasmids and 42 spacers to phages, primarily lysogenic phage ΦEt88 from E. tasmaniensis. This observation may be attributed to the differences in the hosts and niches which these isolates occupy. The E. amylovora isolates which infect apple and pear exist in segregated lifecycles, mainly infecting internal plant material, which minimizes environmental exposure. This may explain the lack of plasmid diversity previously observed within the AI superclade (7), and why most CRISPR spacers provide resistance to plasmids common to E. amylovora, such as pEU30. Comparatively, the isolates of the RI superclade have more diverse CRISPR arrays with an increase in phage targeting spacers suggesting increased exposure to other *Erwinia* spp. and bacterial populations. The majority of *Erwinia* phages have been isolated from soils beneath infected trees (23). Raspberries have higher exposure to soils relative to apple cultivation due to both proximity to the ground and cultivation practices. To limit weeds and disease in raspberry cultivation, the soil between the rows is left bare, or covered with mulch or biogradable plastics. This exposes the *Rubus-*infecting E. amylovora to soil microbiota and their associated phages (24).

### CRISPR-Cas mediated interference in E. amylovora against plasmid pEA-iMGE.

**FIG 2:**
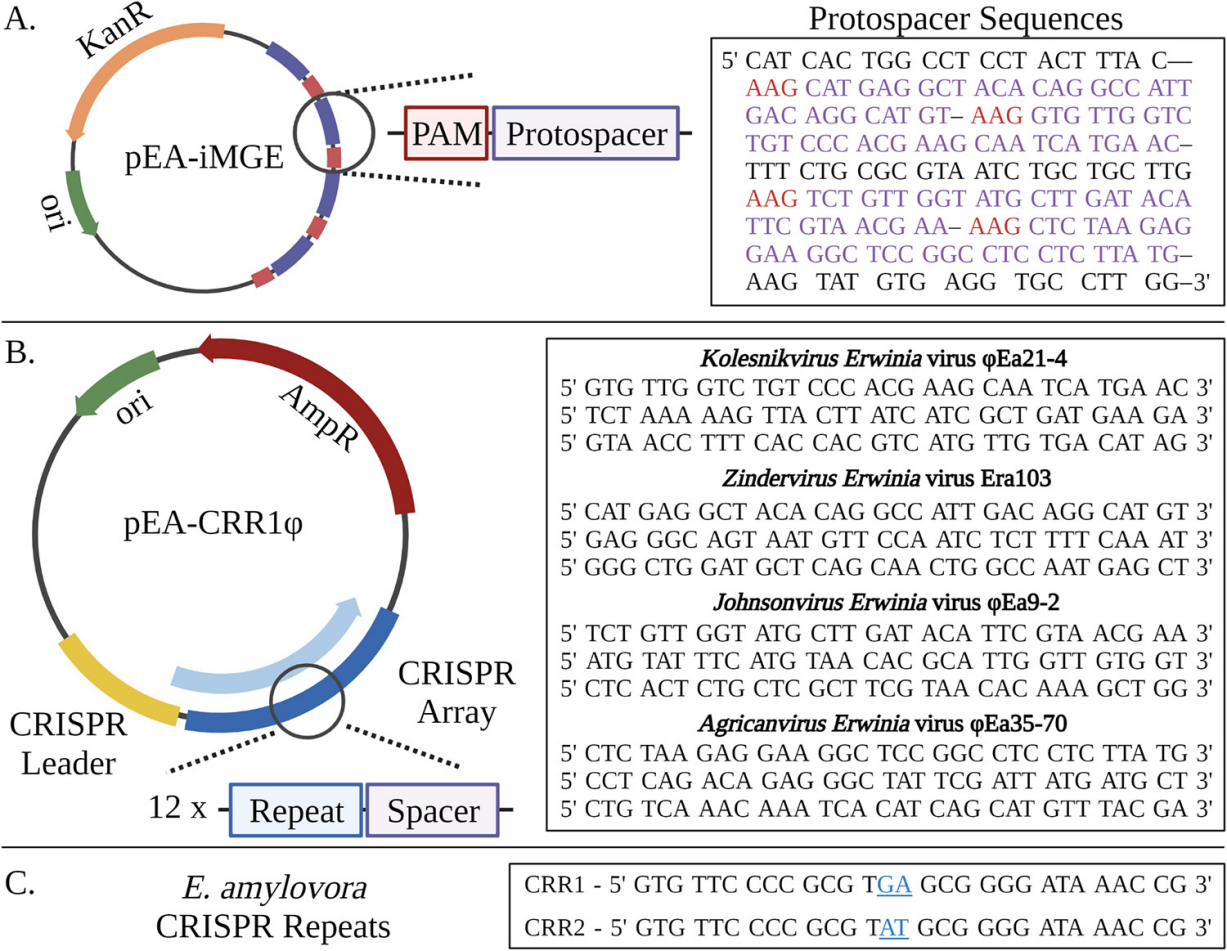
(A) Plasmid pEA-iMGE is a pUC57 plasmid backbone which contains 4 protospacers and the *kanR* gene. PAM and protospacer sequences are highlighted in red and purple, respectively. (B) Plasmid pEA-CRR1Φ is a pUC18 plasmid backbone which contains the ampicillin *ampR* gene in addition to an artificial CRISPR array. The array is composed of the endogenous leader sequence, and the 12 spacer sequences provided, separated by CRISPR repeats. Plasmid pEA-CRR2Φ is identical to pEA-CRR1Φ except the CRISPR repeats and leader sequence were modified to match the CRR2 array of E. amylovora. (C) The CRISPR repeat sequences for CRR1 and CRR2 of E. amylovora with the 2 bp difference highlighted in blue. Created with BioRender.com.

Despite the lack of phage-specific CRISPR spacers, the CRISPR-Cas system of E. amylovora is a potential source of phage resistance which may directly affect the efficacy of phage biocontrol. In previous studies, the plasmid pEU30 has been observed in E. amylovora isolates which also have CRISPR spacers to pEU30 (1, 12). As this plasmid is most prevalent within the WNA clade, the Cas system of CRISPR group III may be compromised. The genomic analysis in this study initially supported this hypothesis as a Q20H mutation in Cas8 was observed in all WNA isolates. Therefore, to determine if the endogenous Cas system was capable of Cascade formation and CRISPR-Cas mediated interference, a two-plasmid system was developed. The first plasmid, pEA-iMGE, contained 4 protospacers designed from the sequences of *Erwinia* phages and conferred kanamycin resistance (Fig. 2A). The second, subsequent transformations were completed with plasmids which conferred ampicillin resistance; pEA-CRR1Φ, pEA-CRR2Φ, and pUC19 as a control. The plasmids pEA-CRR1Φ and pEA-CRR2Φ both contain a 12 spacer CRISPR array, designed with CRR1 and CRR2 repeats, respectively, of which four match the protospacers of pEA-iMGE (Fig. 2B). Consequently, if the isolate is capable of CRISPR-Cas mediated interference, pEA-iMGE will be targeted by the spacers provided by pEA-CRR1Φ or pEA-CRR2Φ, resulting in the loss of kanamycin resistance.

**FIG 3:**
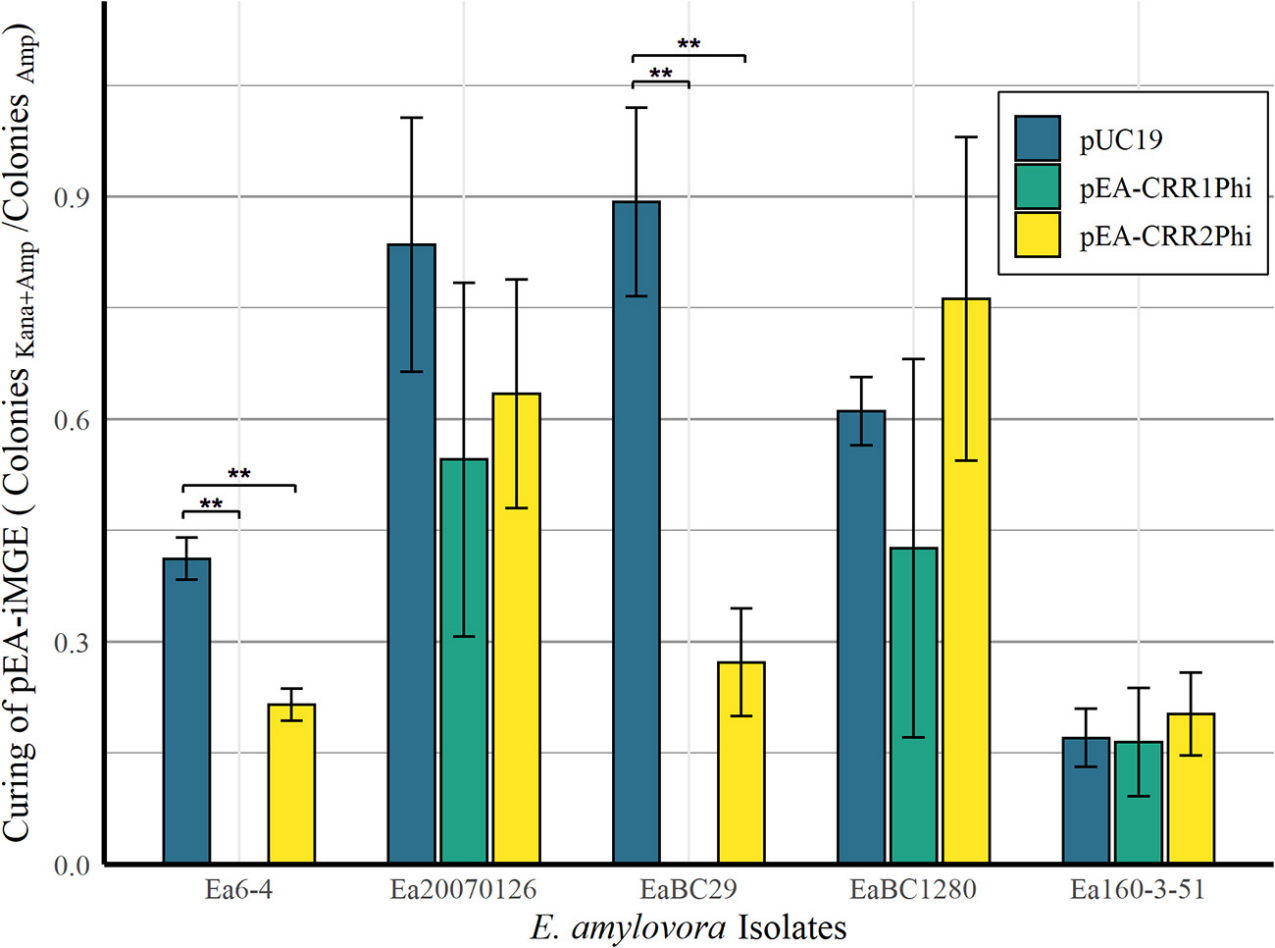
Curing of pEA-iMGE after secondary transformation by pUC19, pEA-CRR1Φ, and pEA-CRR2Φ. Transformations were enumerated after 30 h. Bars represent the mean and the error bars represent a 95% confidence interval of the mean among three replicates. Significance was determined using a Student’s T-test. All significant differences (**) had a *P*-value < 0.01.

E. amylovora CRISPR-Cas activity was first confirmed in WP isolate Ea6-4, which has synonymous Cas genes to all but five WP isolates. In Ea6-4, pEA-CRR1Φ plasmid successfully knocked out pEA-iMGE, whereas pEA-CRR2Φ had a suppressive effect, significantly reducing the relative number of pEA-iMGE harboring transformants (Fig. 3). The difference between the two arrays comes from the leader sequence and a two base pair difference of “GA” to “AT” at position 14 and 15 within the CRISPR repeat (Fig. 2C). As Cas6e both processes and binds the crRNA within Cascade (11), these results suggest the two bp mutation may decrease the affinity of Cas6e to CRR2 repeats. In contrast, the pEU30 harboring WNA isolate 1280, hitherto called EaBC1280, showed no significant change in transformation efficiency for pEA-CRR1Φ or pEA-CRR2Φ relative to the control. In EaBC29, a WNA isolate with the Q20H mutation lacking pEU30, pEA-CRR1Φ and pEA-CRR2Φ were both capable of inducing CRISPR-Cas mediated interference similar to that of Ea6-4 (Fig. 3). Consequently, our hypothesis is that plasmid pEU30, and not the H20Q mutation, is the source of anti-CRISPR activity. All predicted ORFs greater than 30 amino acids were compared to the known anti-CRISPR proteins antagonistic to the Type I-E system (25). While there was some limited homology (<45%) to transcriptional regulators associated with anti-CRISPR activity, such as LuxR and MarR, no significant homology to anti-CRISPR proteins was observed. Simple transformation of pEU30 into E. amylovora to confirm anti-CRISPR activity is not possible due to the size of the plasmid (30 000 bp) and the presence of preexisting CRISPR spacers to pEU30. However, WP Utah isolate Ea20070126, a member of group II, has identical interference-related Cas genes as Ea6-4, lacks the Q20H mutation, and has lost the two CRR1 spacers to pEU30 shared by the WP isolates. It is also the only sequenced WP isolate which harbors plasmid pEU30. Like EaBC1280, Ea20070126 was not capable of CRISPR-Cas mediated interference in the presence of either pEA-CRR1Φ or pEA-CRR2Φ. Lastly, ENA isolate Ea160-3-51 from group IV was incapable of interference, despite having only synonymous mutations within the Cas genes compared to Ea6-4 and Ea20070126. Therefore, while pEU30 is the suspected cause of the decrease in CRISPR-Cas activity, the cause of the lack of CRISPR-Cas activity in Ea160-3-51, and if this cause is shared with the other CRISPR-deficient isolates, remains unknown.

In addition to the strains described above, WP strain ATCC 49946 (Ea273) and RI isolate Ea2-95 were unsuccessfully transformed using the two-plasmid system. While pEA-iMGE and pUC19 could be successfully transformed individually, no viable colonies formed which contained both plasmids resolved to use as a control. E. amylovora strains ATCC 49946 and Ea2-95, harbor pEA72 and pEAR35, respectively, in addition to the ubiquitous pEA29. It is likely that these isolates were incapable of maintaining both the endogenous plasmids as well as adequate copies of the transformed plasmids to provide sufficient antibiotic resistance. Therefore, the applicability of this testing methodology is limited by the number of other endogenous plasmids present in the E. amylovora isolate in question.

### Infection of E. amylovora by phage фEa21-4.

With Ea6-4 confirmed to have CRISPR-Cas activity through plasmid transformation, CRISPR-Cas activity was tested using phage *Kolesnikvirus Erwinia* virus Ea214 (ΦEa21-4). ΦEa21-4 was first isolated on Ea6-4 and is a candidate for phage biocontrol of E. amylovora (26–30). To this end, characteristics about the lytic replication cycle (i.e., adsorption rate, burst size, and time to lysis) have been previously described on this bacterial host through quantitative, real-time PCR (qPCR) (31). This particular form of qPCR utilizes DNase to quantity phage virions, and therefore phage replication, over time. A series of transformants were produced using pEA-CRR1Φ, pEA-CRR2Φ, and pUC19 in the absence of pEA-iMGE. Each of the transformants were then infected with phage ΦEa21-4 and monitored over 8 h using OD_600_ and qPCR.

**FIG 4:**
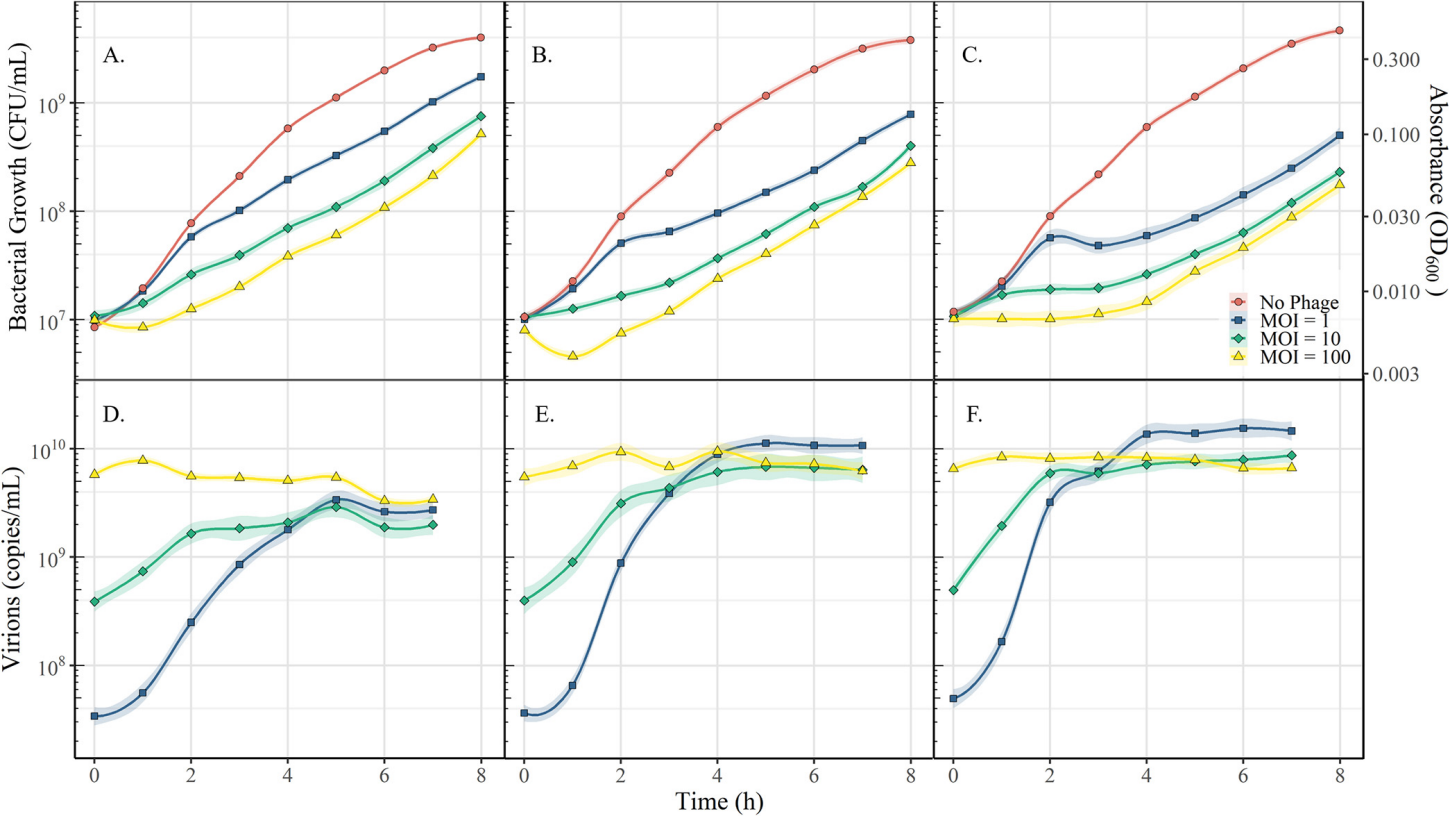
The effect of a targeted CRISPR-Cas response to phage ΦEa21-4 during infection of Ea6-4. Panels (A), (B), and (C) represent the CFU/ml calculated from OD_600_ measures for Ea6-4 transformed by pEA-CRR1Φ, pEA-CRR2Φ, and pUC19, respectively. Panels (D), €, and (F) represent the virion quantification through qPCR for ΦEa21-4 corresponding to (A), (B), and (C), respectively. Red, blue, green, and yellow symbols represent the mean of the triplicate for no phage, MOI of 1, MOI of 10, and MOI of 100, respectively. MOI was confirmed through plaque assay. The line represents the local regression model of the data (LOESS), and the shaded region represents a 95% confidence interval.

Both pEA-CRR1Φ and pEA-CRR2Φ each contain 3 CRISPR spacers capable of targeting ΦEa21-4. The plasmid pEA-CRR1Φ noticeably increased overall growth of the bacterium in the presence of the phage over 8 h by 3.5, 3.3, and 3-fold compared to pUC19 at a multiplicity of infection (MOI) of 1, 10, and 100, respectively (Fig. 4). Overall production of phage decreased 5-fold and quantity of phage present 2 h postinfection was 10-fold less than the pUC19 transformants. This shows that the CRR1 array is able to provide E. amylovora with a degree of immediate phage resistance. Increases in overall growth of pEA-CRR2Φ transformants relative to pUC19 was significantly lower than pEA-CRR1Φ at 56.7% to 75.4%. Likewise, the rate of phage propagation over the first 3 h was reduced by only 14.3%. This exaggerates the observations using the plasmid system in which the CRR1 array provides a higher degree of resistance than CRR2 array. Overall, phage ΦEa21-4 was still able to propagate within Ea6-4 showing the CRISPR-Cas system of E. amylovora does not provide phage immunity, only mild resistance.

While the pEA-CRR1Φ provided immediate protection to phage infection, all phage-infected E. amylovora cultures grew at approximately the same rate (k = 0.4843 to 0.6116) 4 h postinfection regardless of the plasmid present. This is a stark decrease from their uninfected counterparts which grew at rate of k = 0.9586 to 0.9913. The genomic DNA of Ea6-4, infected at an MOI of 100, was extracted and sequenced after 8 h to determine if the ability of pEA-CRR2Φ and pUC19 transformants to grow in the presence of phage ΦEa21-4 was related to CRISPR spacer acquisition. There were no new spacers identified in the genomic assemblies of the endogenous or plasmid-based CRR1 or CRR2 arrays. The raw sequencing reads were also cross referenced against the genome of ΦEa21-4. No new spacers, other than those which were introduced in the artificial CRISPR arrays, were detected in the samples. This indicates that no CRISPR spacer acquisition to ΦEa21-4 occurred and that the CRISPR-Cas system was not responsible for the phage resistance observed in pEA-CRR2Φ and pUC19 transformants. While unexpected, this does agree with the observation that phage growth was decreased but not absent in pEA-CRR1Φ transformants. Lysis of infected hosts is naturally the last step of phage propagation. Therefore, hosts infected by ΦEa21-4, which may have acquired novel spacers, were likely still lysed. This would prevent the development of phage resistance in the E. amylovora population over time.

### Conclusions.

In conclusion, the CRISPR-Cas system of E. amylovora is far more diverse and complex than previous analyses suggested. The phylogenies produced using the CRISPR-Cas system resolve the same clades previously observed in E. amylovora but show a strong connection between the ENA and WNA clades (7, 8). The annotation of the spacers in this work showed that the *Amydaloideae*-infecting strains of E. amylovora are more frequently pressured by plasmids than phages, while the *Rubus*-infecting strains appear to be equally pressured by both. The CRISPR-Cas system is active in the WP and WNA clades in the absence of pEU30, while activity in the ENA clade was not observed. While CRR1 spacers do provide some degree of immediate protection to phage infection, no spacer acquisition to ΦEa21-4 was detected. Interestingly, the control strain of Ea6-4 containing pUC19 was able to survive phage infection using an unidentified system which was complementary to the CRISPR-Cas system. Overall, this shows that while the CRISPR-Cas system is potentially important as a defense mechanism for plasmids, it is not the primary mechanism for phage resistance in *Amydaloideae*-infecting strains of E. amylovora.

## MATERIALS AND METHODS

### Extraction, alignment, and phylogenies of E. amylovora CRISPR arrays.

Genomic sequences for the 127 E. amylovora isolates was accessed through the NCBI nucleotide database as per (7). CRISPR spacers were extracted and aligned from the whole-genome sequences using a pipeline developed in Biopython (this study). CRISPR spacers were obtained through identification of the flanking CRISPR repeats of:

CRR1 (5′-GTGTTCCCCGCGTGAGCGGGGATAACCG-3′).

CRR2 (5′-GTGTTCCCCGCGTATGCGGGGATAAACCG-3′).

CRR4 (5′-GTTCACCTGCCGTACAGGCAGCTTAGAAA-3′).

**FIG 5:**
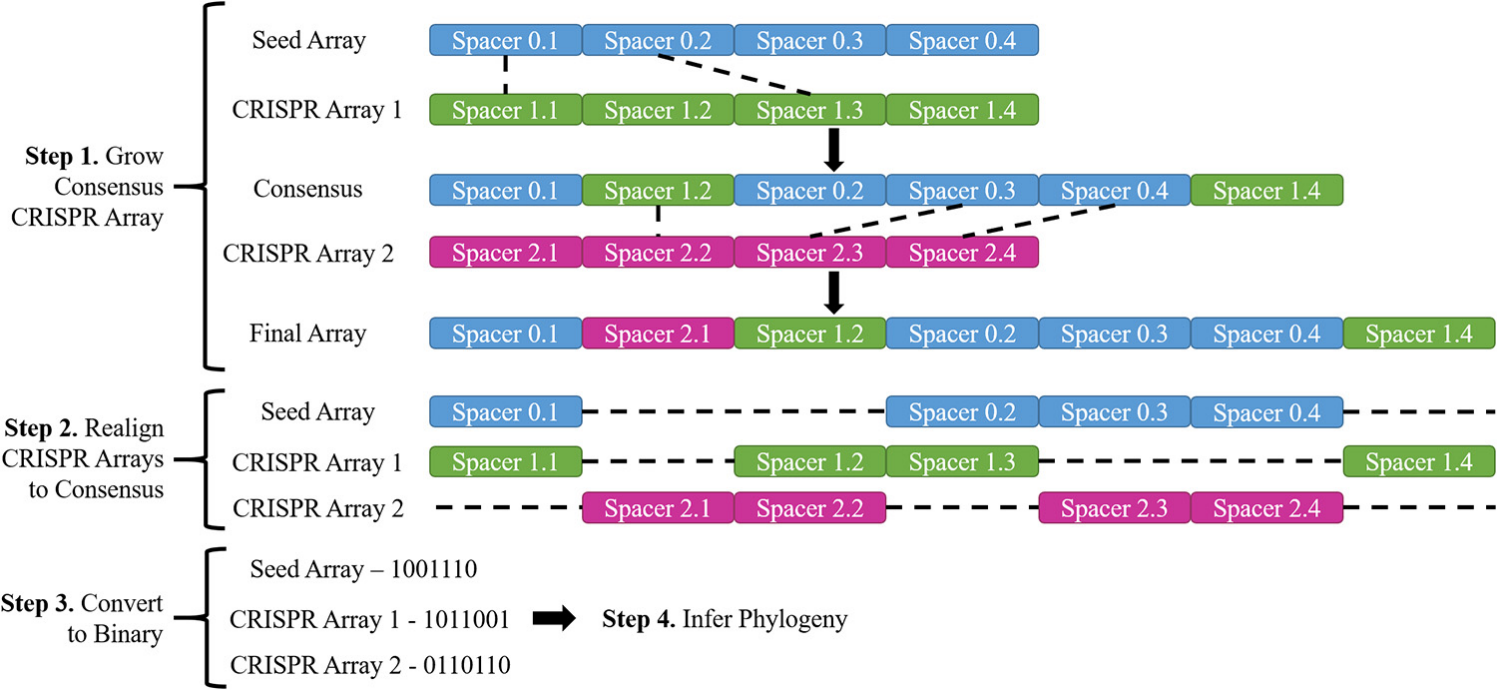
Visual representation of the CRISPR spacer alignment pipeline designed in Biopython. Whole-genome sequences are first parsed to identify CRISPR spacers and form a CRISPR array. The largest CRISPR arrays then acted as a seed, and additional arrays were added to the seed in descending order based on size to form a consensus array. A separate consensus was formed for CRR1, CRR2, and CRR4.

Sequences less than 29 bp, more than 35 bp, or that contained null base calls were excluded due to the high probability of sequencing/assembly errors. All CRISPR spacers were then used to create a consensus alignment for each CRISPR array (CRR1, CRR2, and CRR4). The isolate with the largest CRISPR array, EaOR1, acted as the seed array. The seed array was expanded by sequentially aligning additional CRISPR arrays. Unique spacers were added into the consensus if the flanking spacers were homologous. Otherwise, they were added to the end of the consensus (Fig. 5). After the consensus sequence was completed, the CRISPR arrays were then aligned to the consensus sequence to generate a binary code representing spacers in a array which were congruent with the consensus array. The scripts used to generate the E. amylovora CRISPR consensus sequence is available at github.com/ParceyM/ErwiniaCRISPRAligner. Using the binary sequences which represent the presence and absence of spacers compared to the consensus array, a phylogeny was constructed using IQ-TREE (32) using the GTR2 model, with a bootstrap value of 1000, and a bootstrap cut off 70%. The phylogeny was visualized using iTOL (33).

### Identification of the genetic source of E. amylovora CRISPR spacers.

Isolates were clustered based on the CRISPR group identified within the phylogeny and the CRISPR spacers were pooled. All unique spacers were identified within a given group and compared to the NCBI nucleotide database (34) using a cutoff E value of 1e-3 (identity of approximately 26 of the 32 bp of the spacer). The results were parsed to identify matches to known phages, plasmid, and other iMGEs such as transposons. The plasmid maps identifying the genetic position of the protospacers were created using AngularPlamid (35).

### Design of the artificial CRISPR system and plasmids.

Two artificial CRISPR arrays were synthesized that consisted of the endogenous leader sequences for CRR1 and CRR2 followed by 12 CRISPR spacers within the multiple cloning site of pUC18. The leader sequences were predicted to contain promoters using BPROM (36). The spacers were separated using the CRR1 and CRR2 CRISPR repeats into their own respective plasmids (pEA-CRR1Φ and pEA-CRR2Φ). The CRISPR spacers were designed from the genome of four different genera of *Erwinia* phages (three spacers each): *Kolesnikvirus Erwinia* virus Ea214 (NC_011811.1), *Agricanvirus Erwinia* virus Ea35-70 (NC_023557.1), *Johnsonvirus Erwinia* virus Ea9-2 (KF806588.1), and *Zindervirus Erwinia* virus Era103 (NC_009014.1). The artificial iMGE (pEA-iMGE) consisted of the pUC57-Kan plasmid with one protospacer from each phage for a total of four and separated with an upstream “AAG” PAM sequence (12). qPCR primer/probe target sequences were incorporated at the beginning of synthesized DNA regions to flank the leader sequences. All sequences were synthesized and inserted into vectors by GenScript (Piscataway, NJ, USA).

### Quantification of Ea6-4, pEA-iMGE, pEA-CRR1Φ, pEA-CRR2Φ, and ΦEA21-4.

Random, optimized primers and probes were designed using the genome of *Loxodonta africana* for the detection of pEA-CRR1Φ of pEA-CRR2Φ to ensure no cross-reactivity with E. amylovora (Table 3). Previously developed primers and probes were used for Ea6-4 and ΦEa21-4 (30). In this system, a plasmid containing the Ea6-4 and ΦEa21-4 PCR amplicons was diluted to 10^11^, 10^8^, and 10^5^ copies/mL to create a standard curve for quantification (30). qPCRs contained 2 µL of sample, 200 nM each primer, and 100 nM each probe in EVOlution Probe qPCR mix (Montreal Biotech Inc., Montreal, QC, Canada). Reactions were performed in a qTOWER G3 (Analytik Jena, Jena, Germany) or a Stratagene Mx3005P (Agilent Technologies, Santa Clara, CA, USA) qPCR thermocycler under the following conditions: 15 min at 95°C followed by 40 cycles of 15 s at 95°C and 45 s at 54°C. Prior to the quantification of ΦEa21-4, phage samples were treated with DNase to removed non-encapsidated phage genomes as previously described (31). Briefly, an 8 µL sample of phage was combined with 1 µL of 10x DNase I buffer (B0303D, NEB, Ipswich, MA, USA) and 1 µL DNase I (M0303S, NEB, Ipswich, MA, USA) in a 96-well plate. The samples were then incubated for 40 min at 37°C, followed by 20 min at 95°C, and a hold at 4°C. Phage were also quantified through plaque assays using a soft agar overlay (27, 37).

**TABLE 3:** Primers and probes used for molecular quantification in this study

Target	Name	Sequence
*Erwinia amylovora*	Ea-Lsc-F	CGC TAA CAG CAG ATC GCA
	Ea-Lsc-R	AAA TAC GCG CAC GAC CAT
	Ea-Lsc-P	/5Cy5/CTG ATA ATC CGC AAT TCC AGG ATG/3IAbRQsp/
ΦEa21-4	END37-F	TTC AGC TTT AGC GGC TTC GAG A
	END37-R	AGC AAG CCC TTG AGG TAA TGG A
	END37-P	/56-ROXN/AGT CGG TAC ACC TGC AAC GTC AAG AT/3IAbRQSp/
pEA-CRR1Φ & pEA-CRR2Φ	pEA-CRR-F	CTG GTC AGC ATC ACT AGC ATA A
	pEA-CRR-R	ACC TCG AAG AAG GCG GAT AG
	pEA-CRR-P	/5Cy5/TTT CTG CGC/TAO/GTA ATC TGC TGC TTG/3IAbRQSp/
pEA-iMGE	pEA-iMGE-F	CAT CAC TGG CCT CCT ACT TTA C
	pEA-iMGE-R	CCA AGG CAC CTC ACA TAC TT
	pEA-iMGE-P	/56-FAM/TCC ACT ACG/ZEN/GCC ATC TGT TTC ACG/3IABkFQ/

### Transformation of E. amylovora.

CFU and qPCR copy numbers have been previously correlated to allow CFU/mL to be determined using qPCR for E. amylovora (30). A standard curve for OD_600_ based on qPCR quantification was created using Ea2-95, Ea6-96, and Ea3-97 during exponential growth. A total of 61 measurements were taken at a range of 10^7^ to 10^10^ on a Thermo Spectronic Genesys 20 (ThermoFisher Scientific, Waltham, MA, USA) then quantified through qPCR. From this standard curve, the equation y = 10^10^ · _×1_^.4518^ (R^2^ = 0.9116) was derived where x is the OD_600_ measurement and y is the CFU/mL as determined through qPCR (data not shown).

Isolates were plated on Difco nutrient agar (NA) (BD, Sparks, MD, USA) from frozen cultures stored on microbeads (MicrobankTM, ProLab Diagnostics, Richmond Hill, ON, Canada), incubated at 27°C overnight, and stored at 4°C. A culture of E. amylovora was grown in Difco nutrient broth (BD, Sparks, MD, USA) amended with 0.5% sucrose (NBS), and 100 ppm of kanamycin if required, to 10^8^ CFU/mL in a programmable, Innova 44 shaking incubator (New Brunswick Scientific, Edison, NJ, USA) at 27°C (160 rpm). Following incubation, the bacterial suspension was centrifuged at 12 000 × *g* (4°C) for 8 min and the supernatant was discarded. The bacterial pellet was washed in 40 mL of iced 10% glycerol and centrifuged at 12 000 × *g* (4°C) for 12 min twice. The pellet was resuspended in 10% glycerol and adjusted to 2 × 10^9^ CFU/mL. Each transformation consisted of 400 µL of bacterial suspension and 50 ng of plasmid DNA. Electroporation occurred in 2 mm electroporation cuvettes using a Bio-Rad Gene Pulser Electroporator (Bio-Rad Laboratories, Hercules, CA, USA) with the following settings: 800 Ω, 25 µF, and 2.5 kV for 4s (38). Transformants were immediately diluted with 600 µL of SOC media (39) and incubated at 27°C for 1 h. A 100 µL aliquot of the transformed bacteria was plated on NAS amended with ampicillin, kanamycin or both ampicillin and kanamycin. All antibiotics were applied at 100 ppm.

### CRISPR-Cas mediated interference against plasmids.

Isolates were first transformed with piMGE and were secondarily transformed with pUC19 (control), pEA-CRR1Φ, or pEA-CRR2Φ to test if the introduction of CRISPR spacers homologous to pEA-iMGE resulted in curing of this plasmid. Secondary transformation reactions were plated on NAS amended with ampicillin, kanamycin, or both antibiotics. Efficiency of CRISPR-mediated curing was estimated by the number of colonies on NAS_Kana+Amp_ relative to number of colonies on NAS_Amp_. Transformations were enumerated after 30 h. Transformations were considered to be valid only if growth was observed on NAS_Amp_. Experiments were performed in triplicate. ORFs of pEU30 were identified using ORFfinder and compared to the proteins of the AntiCRISPR Database (AcrDB) (25, 40).

### Propagation and infection using phage ΦEA21-4.

Phage ΦEA21-4 was propagated as previously described with minor amendments (30). Briefly, 100 mL of 10^8^ CFU/mL of the Ea6-4 was prepared in NB and grown at 27°C (160 rpm) in a Innova 44 shaking incubator. After 1 h, 10^8^ PFU of ΦEA21-4 was added to the bacterial culture. The culture was incubated at the conditions listed above overnight. Following incubation, the culture was treated with 2 mL chloroform, centrifuged at 12 000 × *g* (4°C) for 8 min, and passed through a 0.22 µm filter under vacuum (Millipore, Billerica, MA, USA). Phage cultures were stored with 1 mL of chloroform in amber vials (Wheaton Industries, Millville, NJ, USA) at 4°C.

To determine the effect of the CRISPR-Cas system against ΦEA21-4, cultures of Ea6-4 which had been transformed using plasmid pUC19, pEA-CRR1Φ, or pEA-CRR2Φ were grown in NBS amended with 100 ppm ampicillin to 10^8^ CFU/mL as described above. Cultures for OD_600_ sampling were created by diluting the bacterial cultures to 10^7^ CFU/mL in 50 mL falcon tubes with sterile sponge stoppers for aeration. Phage stocks were then diluted and added to the culture at the designated MOI for a total volume of 25 mL. At the same time, a set of paired samples was created at a volume of 150 µL in 96-well plates to quantify phage ΦEA21-4. The infected cultures in 50 mL falcon tubes and sealed 96-well plates were incubated at 27°C (150 rpm) for 8 h. A 1 mL sample was taken from the 25 mL cultures for OD_600_ measurements following phage infection, and every hour thereafter. Fifty µL of chloroform was added to each culture in the paired 96-well plate associated with each time point to kill the culture. Experiments were performed in triplicate. Growth rates were determined using an exponential line of best fit from the equation y = a·e^kt^. The phages were then quantified using the protocol qPCR previously described. After 8 h of incubation, the bacterial genomic DNA was extracted from the cultures infected at an MOI of 100 using the Bacterial Genomic DNA isolation kit (17900, Norgen Biotek Corp., St. Catharines, ON, Canada) as per the manufacturer’s instructions.

### Sequencing of the Ea6-4 transformants infected by phage ΦEA21-4.

The extracted genomic DNA was sequenced using the Nanopore MinIon platform (Oxford Nanopore Technologies, Oxford, UK). Samples were prepared using the manufacture’s instructions for the Rapid DNA Sequencing kit (SQK-RAD004, Oxford Nanopore Technologies, Oxford, UK) on a Spoton flow cell (Oxford Nanopore Technologies, Oxford, UK). Sequencing data were acquired using MinKnow and the genomes were assembled using Flye with 4 polishing steps (41). Coverage of each assembly was at least 50x. The chromosomal and plasmid sequences were assessed for the insertion of new CRISPR spacers using the CRISPR aligner pipeline. The sequencing data were also parsed to identify individual reads which contained any CRISPR repeats. The reads were then cross-referenced to the phage ΦEA21-4 genome using blastn to determine if any novel spacers had been acquired which didn’t appear in the genomic assemblies (42).
